# 

**Published:** 1855-11

**Authors:** 


☞ Messrs. A. TILDEN and W. H. PHILLPOT are authorized Agents for this Journal.

---

**PRIVATE MEDICAL INSTRUCTION.**

THE undersigned design to give a Course of Private Instruction to such Medical Students as may desire to enter their office. Regular lectures and examinations on the various branches of medical science will be afforded twice a week.

J. G. WESTMORELAND, M. D.,

ATLANTA, Sept. 1, 1855. W. F. WESTMORELAND, M. D.

